# Integrating Single‐Cell Transcriptomics and Machine Learning to Define an ac4C Gene Signature in Lung Adenocarcinoma

**DOI:** 10.1111/1759-7714.70140

**Published:** 2025-08-06

**Authors:** Yuan Wang, Wei Su, Guangyao Zhou, Yijie Wang, Chunnuan Wu, Pengpeng Zhang, Lianmin Zhang

**Affiliations:** ^1^ Key Laboratory of Cancer Prevention and Therapy Tianjin Medical University Cancer Institute and Hospital, National Clinical Research Center for Cancer, Tianjin's Clinical Research Center for Cancer Tianjin China; ^2^ Department of Lung Cancer, Tianjin Lung Cancer Center, National Clinical Research Center for Cancer, Key Laboratory of Cancer Prevention and Therapy Tianjin's Clinical Research Center for Cancer, Tianjin Medical University Cancer Institute and Hospital Tianjin China; ^3^ Department of Endoscopy Tianjin Medical University Cancer Institute and Hospital, National Clinical Research Center for Cancer Tianjin China

**Keywords:** lung cancer, machine learning, N4‐acetylcytidine, prognostic signature, RNA modification

## Abstract

**Introduction:**

Lung adenocarcinoma, the most common subtype of non‐small cell lung cancer, faces challenges such as drug resistance and tumor heterogeneity. N4‐acetylcytidine (ac4C) is an important RNA modification involved in cancer progression, but its role in lung adenocarcinoma remains unclear.

**Methods:**

This study analyzed transcriptomic and single‐cell RNA sequencing data from public databases to investigate the expression and clinical significance of ac4C‐related genes in lung adenocarcinoma. Ten machine learning algorithms were applied to develop and validate an ac4C‐related gene signature (ARGSig) for prognosis prediction across multiple independent cohorts.

**Results:**

Cells with high ac4C activity showed increased intercellular communication and activation of tumor‐associated pathways. The ARGSig model effectively stratified patients by survival outcomes and predicted sensitivity to immune checkpoint inhibitors and chemotherapy agents.

**Conclusion:**

ac4C modification and its related genes play a critical role in lung adenocarcinoma development. The ARGSig model provides a promising molecular tool for prognosis evaluation and personalized treatment guidance in lung adenocarcinoma patients.

## Graphical abstract

This study combines single‐cell RNA sequencing and bulk transcriptomics to investigate the role of ac4C RNA modification in lung adenocarcinoma. Using 10 machine learning algorithms, an ac4C‐related gene signature (ARGSig) was constructed and validated for prognosis prediction.

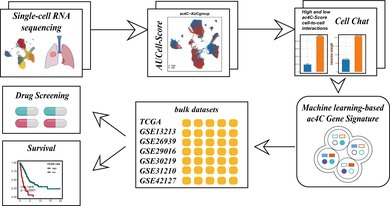

**Abbreviations**
ac4C: N4‐acetylcytidine
ARGSig: ac4C‐related gene signature
AUC: area under the dose–response curve
CCLE: Cancer Cell Line Encyclopedia
C‐index: concordance index
CNV: copy number variation
DEGs: differentially expressed genes
EMT: epithelial–mesenchymal transition
FDR: false discovery rate
GEO: Gene Expression Omnibus
GSVA: gene set variation analysis
ICIs: immune checkpoint inhibitors
IPS: immunophenoscores
LUAD: lung adenocarcinoma
ML: machine learning
MSigDB: Molecular Signatures Database
NSCLC: non‐small cell lung cancer
OS: overall survival
PCA: principal component analysis
ROC: receiver operating characteristic
RSF: random survival forest
SCLC: small cell lung cancer
scRNA‐seq: single‐cell RNA sequencing
ssGSEA: single‐sample gene set enrichment analysis
survivalSVM: survival support vector machine
TCGA: The Cancer Genome Atlas
TMB: tumor mutation burden
TPM: transcripts per million
UMAP: Uniform Manifold Approximation and Projection
WHO: World Health Organization

## 1 Introduction

Lung cancer is the leading cause of cancer‐related mortality in both sexes and represents the most common malignant tumor within the respiratory system [1]. According to histological classification, the World Health Organization (WHO) categorizes lung malignancies into non‐small cell lung cancer (NSCLC) and small cell lung cancer (SCLC) [2]. NSCLC accounts for approximately 85% of cases and is the predominant form of lung cancer [3]. Lung adenocarcinoma, the most frequent subtype of NSCLC, constitutes about 40% of NSCLC cases [4, 5]. Surgical intervention remains the most effective treatment for early‐stage lung adenocarcinoma patients (stages I and II) [6]. However, the majority of patients are diagnosed at advanced stages, rendering complete surgical resection challenging. Over the past decade, significant therapeutic advancements have been achieved through the development of molecular targeted therapies and immunotherapy. Immune checkpoint inhibitors (ICIs) have notably improved clinical outcomes in lung adenocarcinoma and represent a promising option for neoadjuvant immunotherapy in early resectable disease [7]. Nevertheless, studies indicate that the 5‐year survival rate remains low at 4%–10%, benefiting only a minority of patients [8, 9, 10]. Molecular targeted therapy and immunotherapy continue to face substantial obstacles, primarily drug resistance and increased toxicity. Combination treatments, in particular, often induce severe adverse effects that frequently necessitate treatment discontinuation. Furthermore, the high heterogeneity of lung adenocarcinoma contributes to diverse mechanisms of resistance among patients, likely representing a major factor in therapeutic failure [11]. Therefore, there is an urgent need to elucidate the underlying molecular mechanisms and develop robust molecular classification models to accurately assess prognosis and guide personalized therapeutic strategies for lung adenocarcinoma (LUAD) patients.

RNA modification constitutes a fundamental component of epigenetic regulation [12]. It not only participates in gene expression control and cellular fate determination but also exerts a profound influence on tumor initiation, progression, and recurrence [13]. Increasing evidence indicates that dysregulation of RNA modifications can contribute to oncogenesis, presenting potential targets for cancer therapy [14]. N4‐acetylcytidine (ac4C) is a conserved nucleoside modification present on tRNA and rRNA, representing a chemical alteration of RNA [15]. As an mRNA modification, ac4C plays a critical role in maintaining mRNA stability and enhancing translation. Specifically, under physiological conditions, ac4C is catalyzed enzymatically by the addition of an acetyl group to the N4 position of cytidine, affecting RNA stability, structure, and translational efficiency. Beyond normal physiology, ac4C modification exerts regulatory functions in various cancers and has been implicated in processes including cancer cell proliferation, metastasis, metabolism, and chemoresistance. In esophageal cancer, ac4C is significantly upregulated and associated with poor prognosis [16]. Studies in gastric cancer have shown that inhibiting ac4C reduces the hypoxia tolerance of cancer cells, thereby suppressing tumor progression in vivo [17]. NAT10 is currently identified as the sole enzyme responsible for catalyzing this modification. In prostate cancer, the acetyltransferase NAT10 promotes ac4C synthesis, facilitating epithelial–mesenchymal transition (EMT) and enhancing cellular migration [18]. Given its differential expression and pivotal role in cancer progression, ac4C represents a promising target for tumor diagnosis and therapy. However, the relationship between ac4C modification and lung adenocarcinoma remains insufficiently explored.

This study systematically evaluates the role of ac4C modification in lung adenocarcinoma using single‐cell RNA sequencing (scRNA‐seq) to dissect the cellular composition of LUAD patients and its association with ac4C modification activity. Based on differentially expressed genes (DEGs) within cellular subpopulations, we developed a predictive model employing 10 machine learning algorithms, achieving superior accuracy compared to previous models and demonstrating potential clinical applicability. Functionally, we further assessed the model's capability to predict responses to immunotherapy and chemotherapy. Collectively, this research offers novel insights into the application of ac4C modification in lung adenocarcinoma and provides a foundation for personalized treatment and improved patient prognosis.

## 2 Methods

### 2.1 Data Sources

This study obtained transcriptomic and copy number variation (CNV) data for lung adenocarcinoma (LUAD) from The Cancer Genome Atlas (TCGA) database (https://portal.gdc.cancer.gov) to serve as the training set for model development. For model validation, six transcriptomic datasets were retrieved from the Gene Expression Omnibus (GEO) database: GSE13213 (*n* = 119) [19], GSE26939 (*n* = 115) [20], GSE29016 (*n* = 39) [21], GSE30219 (*n* = 86) [22], GSE31210 (*n* = 227) [23], and GSE42127 (*n* = 134) [24]. Additionally, two single‐cell RNA sequencing datasets were sourced from GEO (GSE189357) [25] and the Genome Sequence Archive (HRA001130) [26].

To ensure data consistency and comparability, all gene expression profiles were converted to transcripts per million (TPM) format. The “combat” function from the R package “sva” was applied to mitigate potential batch effects [27]. Moreover, all TCGA and GEO datasets were log‐transformed to standardize data formatting. Principal component analysis (PCA) was conducted to evaluate batch effects among datasets.

### 2.2 Identification of Prognosis‐Related ac4C Genes

Differentially expressed ac4C‐related genes between normal and tumor tissues were identified using the limma package, with criteria set as false discovery rate (FDR) < 0.05 and |log2 fold change (FC)| > 1. Univariate Cox regression analysis was subsequently performed to assess the prognostic significance of these genes in LUAD patients. Based on 10‐fold cross‐validation, a comprehensive model selection was conducted using 10 machine learning algorithms—stepwise Cox regression, Lasso, Ridge, partial least squares regression for Cox models (plsRcox), CoxBoost, random survival forest (RSF), generalized boosted regression model (GBM), elastic net (Enet), supervised principal components (SuperPC), and survival support vector machine (survival‐SVM)—resulting in 101 model combinations. The optimal ac4C‐related gene signature (ARGSig) was selected according to the highest concordance index (C‐index). Our approach was adapted from Liu et al., who utilized similar algorithms but employed leave‐one‐out cross‐validation; here, 10‐fold cross‐validation was adopted [28]. Model accuracy was evaluated by receiver operating characteristic (ROC) curves and PCA. Gene set variation analysis (GSVA) was employed to explore underlying biological mechanisms. Immune cycle and immunotherapy response‐related pathways were assessed according to established protocols, with pathway gene sets obtained from the Molecular Signatures Database (MSigDB).

### 2.3 Integrated Analysis of Genomic Variations and Immune Cell Infiltration

GISTIC 2.0 was utilized to identify recurrent genomic amplifications and deletions. Tumor mutation burden (TMB) was calculated using the R package “maftools.” Tumor immune gene expression and immune cell infiltration data were acquired from the TIDE website (http://tide.dfci.harvard.edu/), The Cancer Immunome Atlas (https://tcia.at/home), TIMER2.0 database, and the IOBR R package. Immune cell abundance and pathway activity were quantified using the single‐sample gene set enrichment analysis (ssGSEA) algorithm.

### 2.4 Single‐Cell Gene Expression Data Preprocessing and Integration

Preprocessing was performed using the Seurat package (version 4.2.0). Genes expressed in at least 10 cells were retained. Cells expressing fewer than 200 or more than 5000 genes, or exhibiting mitochondrial gene expression exceeding 10%, were excluded. Sample integration was conducted via the Harmony package. Highly variable genes were selected for principal component analysis (PCA), with the top 30 components retained. Uniform Manifold Approximation and Projection (UMAP) was applied for dimensionality reduction and visualization. DEGs within cell subpopulations were identified using the “FindAllMarkers” function, and cell types were annotated based on known marker genes.

### 2.5 Cell–Cell Communication Analysis

CellChat was employed to integrate gene expression data and analyze differences in intercellular communication. The default CellChatDB ligand–receptor database was utilized. Overexpressed ligands or receptors were detected to infer cell‐specific interactions and identify enhanced ligand–receptor pairs.

### 2.6 Drug Sensitivity Analysis in Cancer Cell Lines and LUAD Patients

Human cancer cell line expression data were sourced from the Broad Institute's Cancer Cell Line Encyclopedia (CCLE). CERES scores, reflecting gene dependency for cell survival and proliferation, were obtained from the DepMap portal, with lower scores indicating higher gene essentiality. Drug sensitivity data were derived from the CTRP and PRISM databases and quantified by the area under the dose–response curve (AUC), where lower AUC values represent greater sensitivity. Missing values were imputed using the k‐nearest neighbors method; compounds and hematopoietic or lymphoid lineage cell lines with over 20% missing data were excluded. The pRRophetic package was applied, integrating drug sensitivity and genomic datasets to predict chemotherapy response in LUAD patients based on ridge regression models.

### 2.7 Statistical Analysis

All data processing and statistical analyses were conducted in R software (version 4.2.0). Overall survival (OS) differences among subgroups were assessed using Kaplan–Meier survival curves and log‐rank tests. Comparisons of continuous variables between two groups were performed using Wilcoxon rank‐sum tests or Student's *t* tests, whereas categorical variables were compared via *χ* ^2^ tests or Fisher's exact tests. Multiple testing correction employed the FDR method. Pearson correlation coefficients were calculated to evaluate variable associations. All statistical tests were two‐sided, with *p* values less than 0.05 considered statistically significant.

## 3 Results

### 3.1 Cell Type Identification and Marker Gene Screening

**FIGURE 1:**
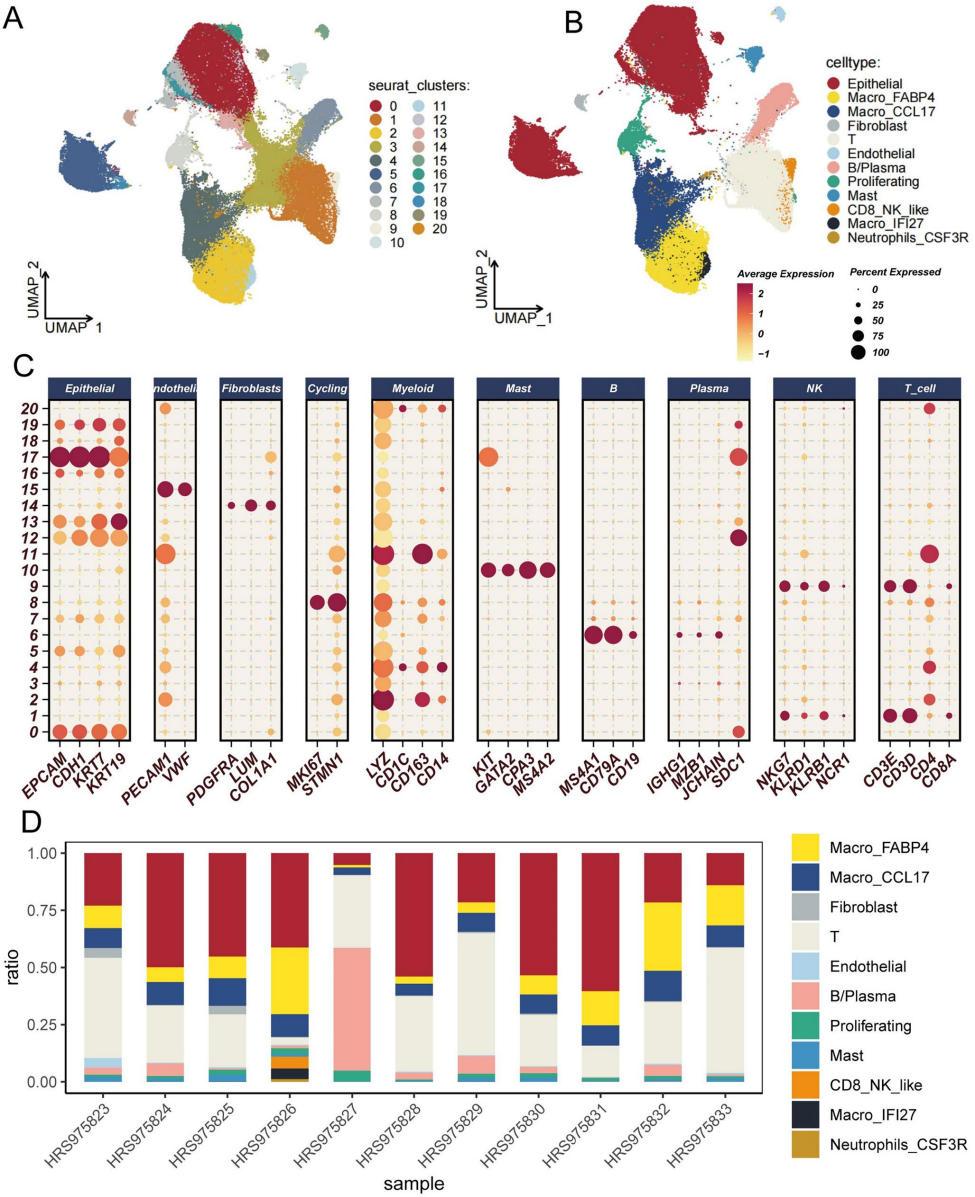
Cell type annotation based on distinct cell markers in lung cancer samples. (A) UMAP visualization of 21 clusters. (B) UMAP visualization of 12 cell types, including Epithelial, Macro_FABP4, Macro_CCL17, Fibroblast, T, Endothelial, B/Plasma, Proliferating, Mast, CD8_NK_like, Macro_IFI27, Neutrophils‐CSF3R. (C) The dot plot illustrates the average and percent expression levels of marker genes across various cell subtypes. (D) The line chart displays the distribution of each cell type throughout the samples.

**FIGURE 2:**
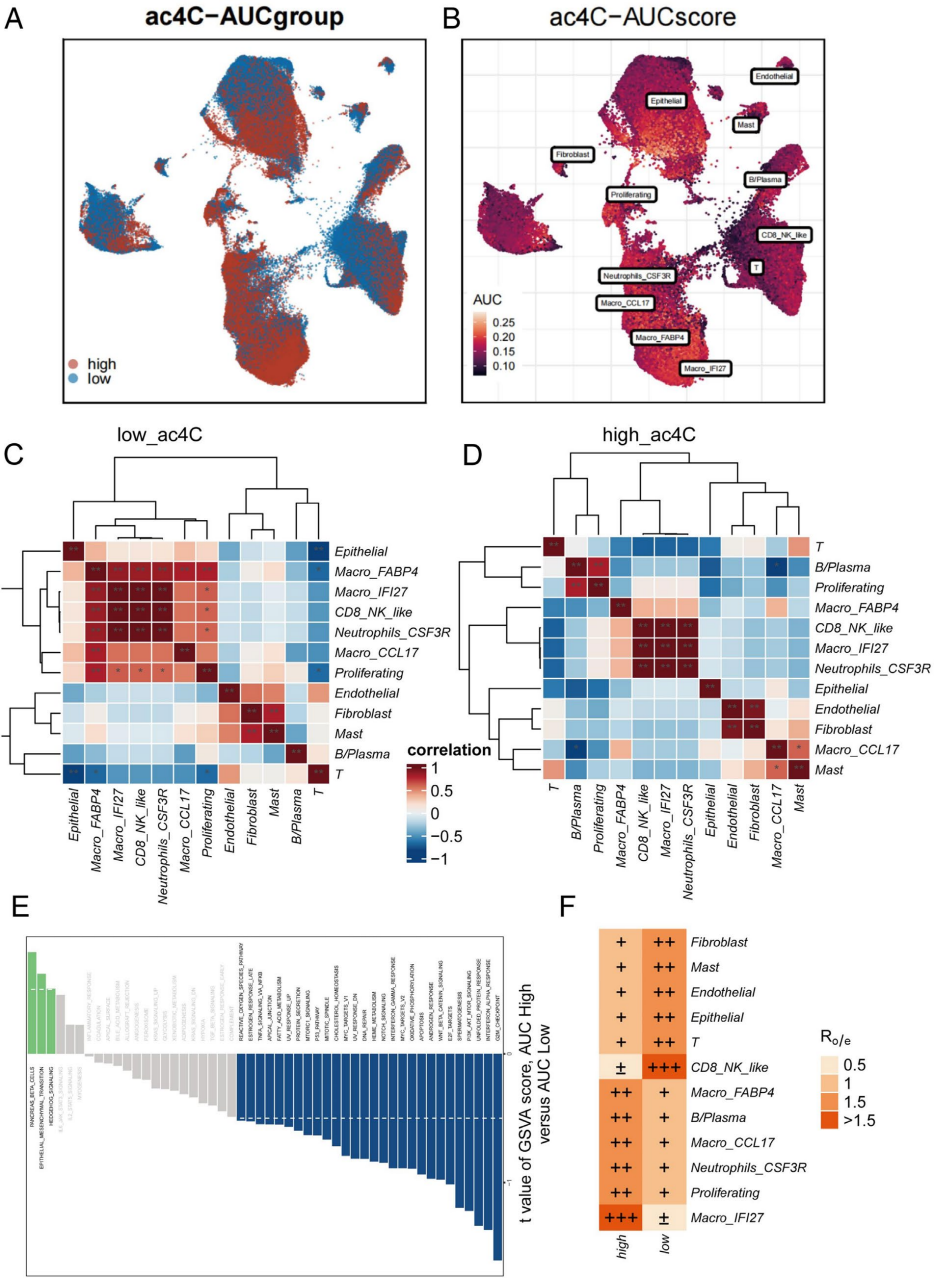
Similarities and differences between ac4C‐high and ac4C‐low cells. (A) The distribution of high‐score cells and low‐score cells. (B) Scores of ac4C‐related genes in clusters, by AUCell. (C) The correlation of cell clusters in ac4C‐low cells. (D) The correlation of cell clusters in ac4C‐high cells. (E) The difference of GSVA scores. (F) The enrichment of ac4C‐high and ac4C‐low cells in different cell clusters, by single‐cell tissue preference analysis.

We obtained a publicly available single‐cell RNA sequencing dataset from the GEO database to analyze cellular populations from various non‐small cell lung cancer patients. After dimensionality reduction, 21 distinct cellular subpopulations were identified (Figure 1A). Based on specific gene expression patterns within these clusters, cell types were annotated as epithelial, Macro_FABP4, Macro_CCL17, fibroblast, T cells, endothelial, B/plasma, proliferating, mast cells, CD8_NK_like, Macro_IFI27, and Neutrophils_CSF3R (Figure 2B,C). Additionally, 11 lung adenocarcinoma samples were obtained from HRA005794, with the percentage distribution of cell types per sample depicted in Figure 2E. Guided by an extensive literature review, genes most closely associated with ac4C modification were selected for further analysis. Using the AUCell algorithm, cells were scored and subsequently divided into high (ac4C‐high) and low (ac4C‐low) scoring groups based on the median score. The results indicated that Epithelial and Macro_FABP4 cells exhibited higher ac4C‐related gene activity, whereas T cells showed lower scores, suggesting differential ac4C gene expression activity among these populations (Figure 2A,B).

### 3.2 Transcriptomic Similarity and Differences Between ac4C‐High and ac4C‐Low Cells

Correlation analyses and clustering generated heatmaps revealing that in ac4C‐low cells, Macro_FABP4 and Macro_CCL17, CD8_NK_like, Neutrophils_CSF3R, Macro_IFI27, and Proliferating cells demonstrated strong transcriptomic correlations (*p* < 0.01). Proliferating cells showed moderate correlation with Macro_IFI27, CD8_NK_like, Neutrophils_CSF3R, and Mast cells (*p* < 0.05). Conversely, in ac4C‐high cells, significant correlations were observed between B/Plasma and Proliferating cells, CD8_NK_like with Macro_IFI27 and Neutrophils_CSF3R, and endothelial with fibroblast populations (*p* < 0.01). Moderate correlations were found between NK/T cells and T regulatory cells (*p* < 0.05) (Figure 2C,D). To explore differences between ac4C‐high and ac4C‐low cells, canonical gene sets from the Broad Institute were applied. Compared to ac4C‐low cells, ac4C‐high cells showed significant upregulation of tumor‐associated pathways, including metabolic processes and epithelial‐mesenchymal transition, with three pathways upregulated, 28 downregulated, and 19 unchanged (Figure 2E).

Single‐cell tissue preference analysis revealed significant enrichment of Macro_IFI27, Macro_FABP4, B/Plasma, Macro_CCL17, and Proliferating cells in the ac4C‐high group, particularly Macro_IFI27, while CD8_NK_like cells were diminished. In contrast, CD8_NK_like, T cells, epithelial, endothelial, mast cells, and fibroblasts were enriched in the ac4C‐low group, where Macro_IFI27 was reduced (Figure 2E).

### 3.3 Differences in Cell–Cell Communication Between Groups

**FIGURE 3:**
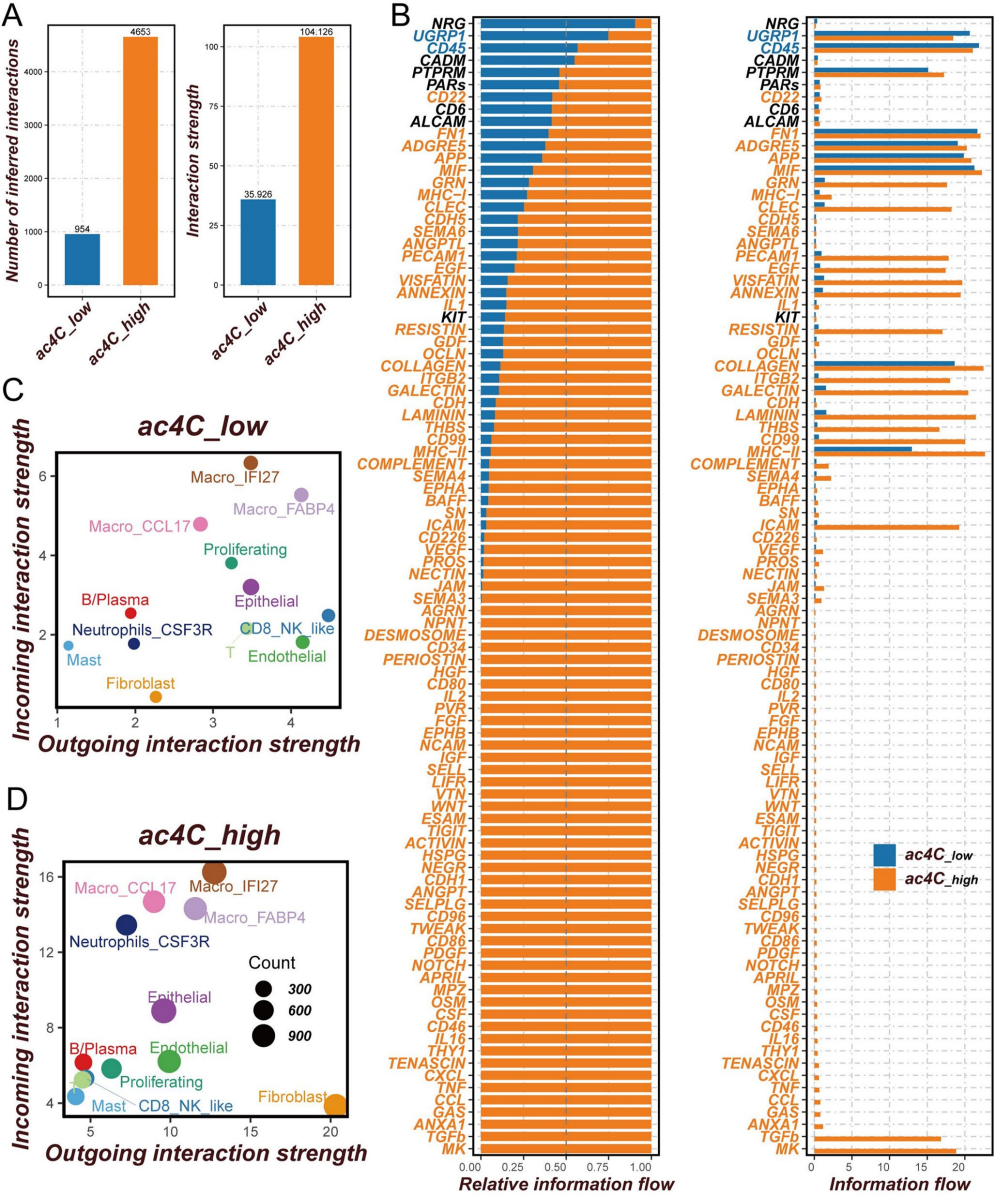
Cell–cell communication analysis between ac4C‐high and ac4C‐low cells. (A) Number of inferred interactions and interaction strength in ac4C‐high and ac4C‐low cells. (B) Information flow in ac4C‐high and ac4C‐low cells. (C) Interaction strength of 12 cell clusters in ac4C‐high cells. (D) Interaction strength of 12 cell clusters in ac4C‐low cells.

Cell–cell communication analysis demonstrated that ac4C‐high cells exhibited significantly increased numbers and intensities of intercellular interactions compared to ac4C‐low cells (Figure 3A). To identify key signaling pathways driving these differences, information flow between ac4C‐high and ac4C‐low cells was compared. Pathways including PTPRM, PARs, CD6, ALCAM, CD22, and MIF were active in both groups without significant differences. However, signaling via GRN, CLEC, PECAM1, EGF, VISFATIN, and ICAM pathways was markedly upregulated in ac4C‐high cells (Figure 3B). All 12 cellular subtypes in the ac4C‐high group exhibited enhanced communication, indicative of more active intercellular interactions. Among these, Fibroblasts showed the highest outbound interactions, whereas Macro_IFI27 cells displayed the highest inbound communication. Fibroblasts engaged robustly with the other 11 cell types, predominantly epithelial cells, endothelial cells, and macrophages, with these interactions being more pronounced in ac4C‐high cells relative to ac4C‐low cells (Figure 3C,D). Previous research has elucidated the role of ac4C in modulating the function of macrophages within the tumor microenvironment. In esophageal squamous cell carcinoma, NAT10 stabilizes the expression of fatty acid synthase (FASN) by mediating ac4C modification, thereby promoting the M2 polarization of macrophages [29]. In nasopharyngeal carcinoma, the inflammatory macrophage apoptosis inhibitor lncRNA (SIMALR) is highly expressed in tumor tissues. NAT10 enhances the stability of SIMALR through ac4C modification, which in turn facilitates the proliferation and metastasis of nasopharyngeal carcinoma cells [30]. Regarding NSCLC, the stability of GAS5 is regulated by ac4C modification. Elevated expression of GAS5 promotes the recruitment of macrophages and T cells, thereby inhibiting the progression of NSCLC [31]. In the future, we need to conduct more systematic and comprehensive studies on the role of ac4C in other cell subpopulations.

### 3.4 Elucidation of ac4C‐Associated Biomarkers

**FIGURE 4:**
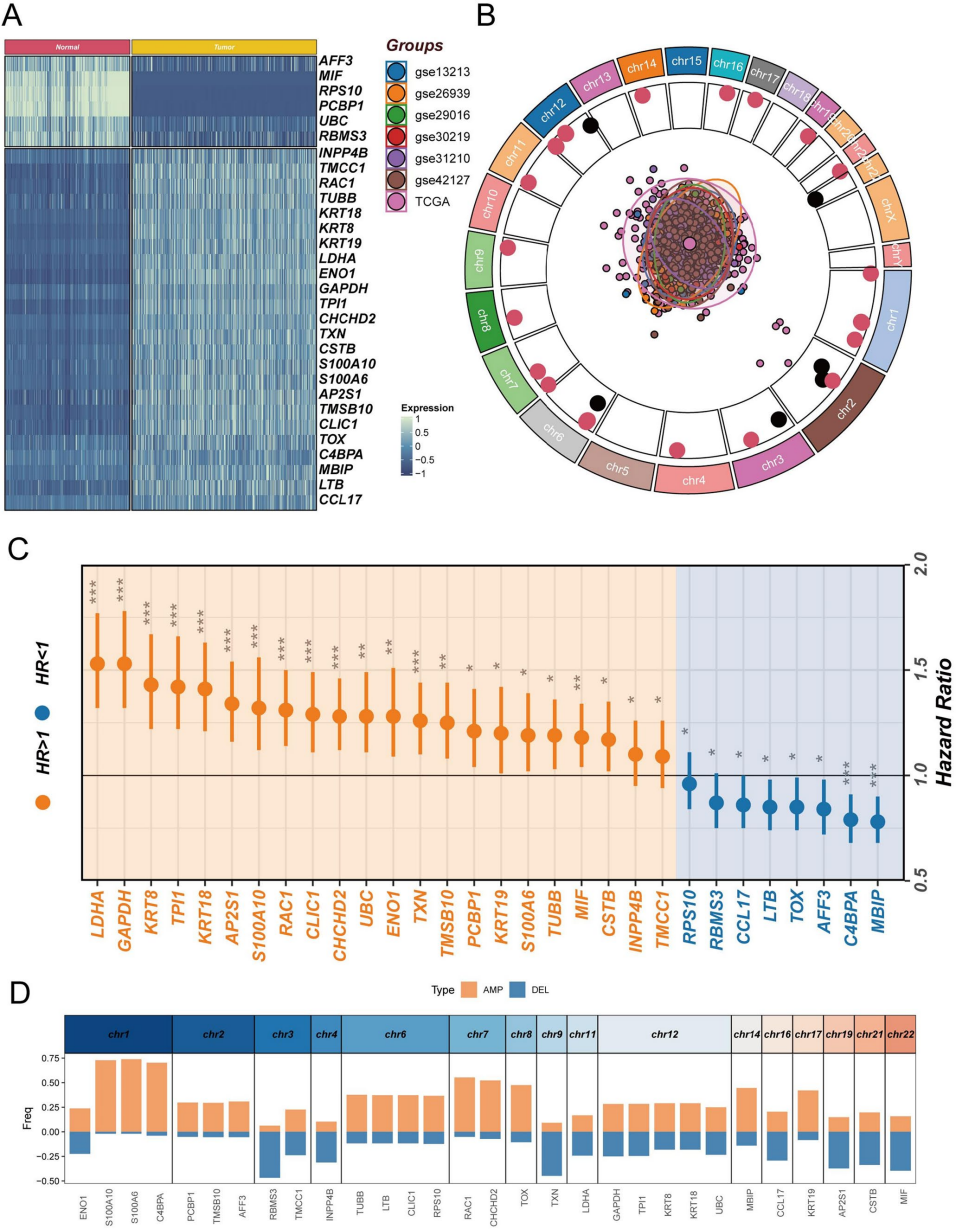
Identification and analysis of prognostic ac4C‐related genes. (A) Identification of differentially expressed ac4C‐related genes in tumor compared with normal lung tissue. (B) Chromosomal locations of prognostic ac4C‐related genes, with red dots representing higher expression in tumors relative to normal lung tissue (depicted by log2FC values; the larger the value, the more peripheral the position), and black dots representing the opposite. The central pie chart shows all LUAD datasets used for modeling and principal component analysis plot after batch effect removal in seven LUAD datasets. (C) Univariate Cox analysis to determine potential prognostic ac4C‐related genes. (D) Chromosomal locations and copy number variations of prognostic ac4C‐related genes.

To deepen understanding of ac4C's role in cancer, we identified a panel of ac4C‐related biomarkers with specific focus on LUAD. Differential expression analysis integrating normal lung tissue data from GTEx and LUAD samples from TCGA revealed differentially expressed ac4C‐associated genes (Figure 4A). Batch effects were mitigated across seven LUAD transcriptomic cohorts to enhance comparability (Figure 4B). Univariate Cox regression identified ac4C genes significantly associated with prognosis (Figure 4C). CNV analysis indicated frequent genomic alterations in ac4C‐related genes, with S100A10 and S100A6 exhibiting the most extensive CNV amplifications (Figure 4D).

### 3.5 Model Development, Validation, and Clinical Applicability for Immunotherapy and Chemotherapy

**FIGURE 5:**
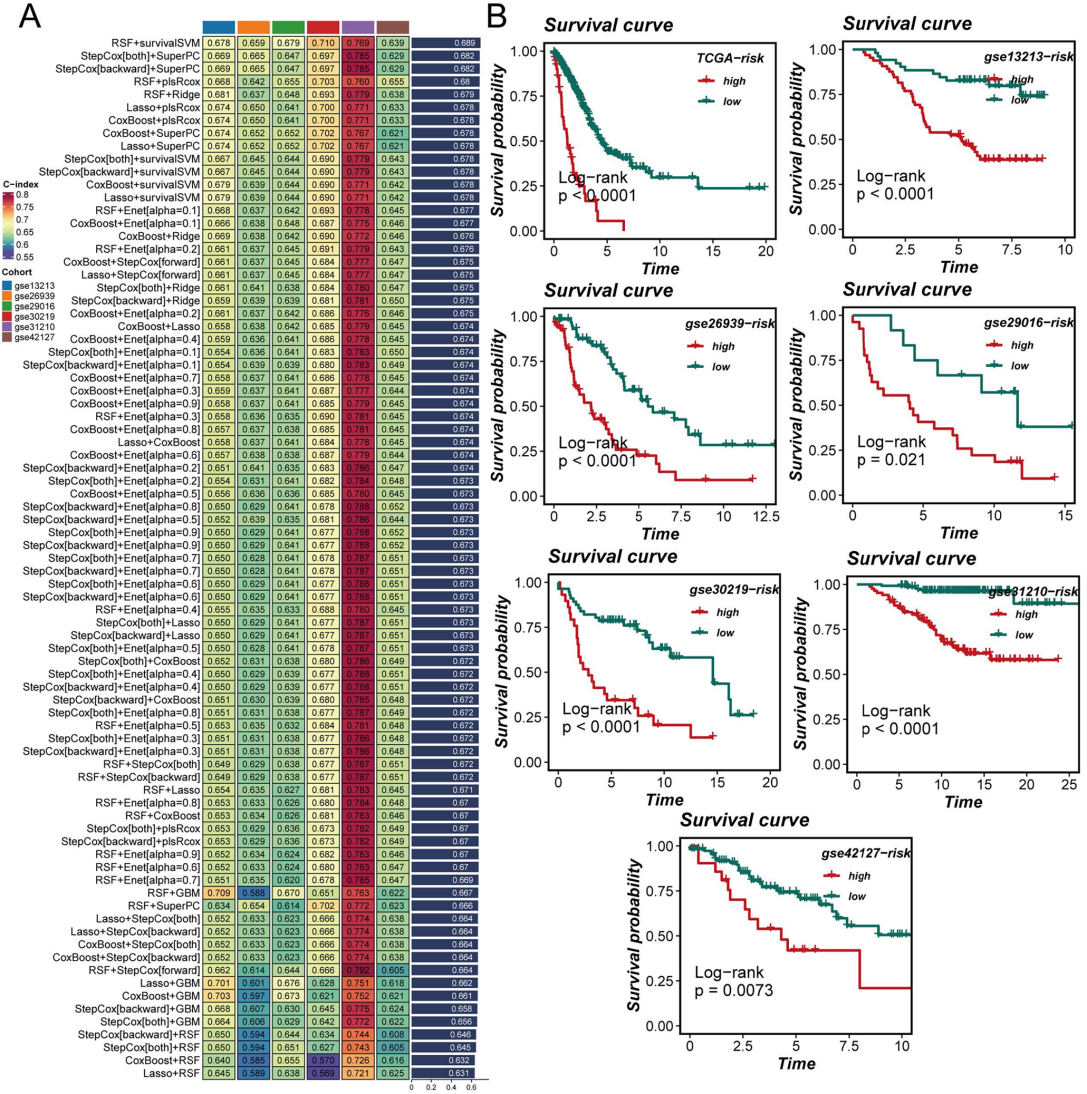
Construction and validation of ac4C‐related genes using machine learning. (A) Construction of ac4C‐related genes using various machine learning combinations, with values in the heatmap representing the C‐index of corresponding models for predicting prognosis; the bar graph on the right shows the average C‐index across multiple datasets. (B) ac4C‐related genes effectively stratify prognosis in LUAD patients across seven datasets, with the low ac4C group showing better outcomes.

**FIGURE 6:**
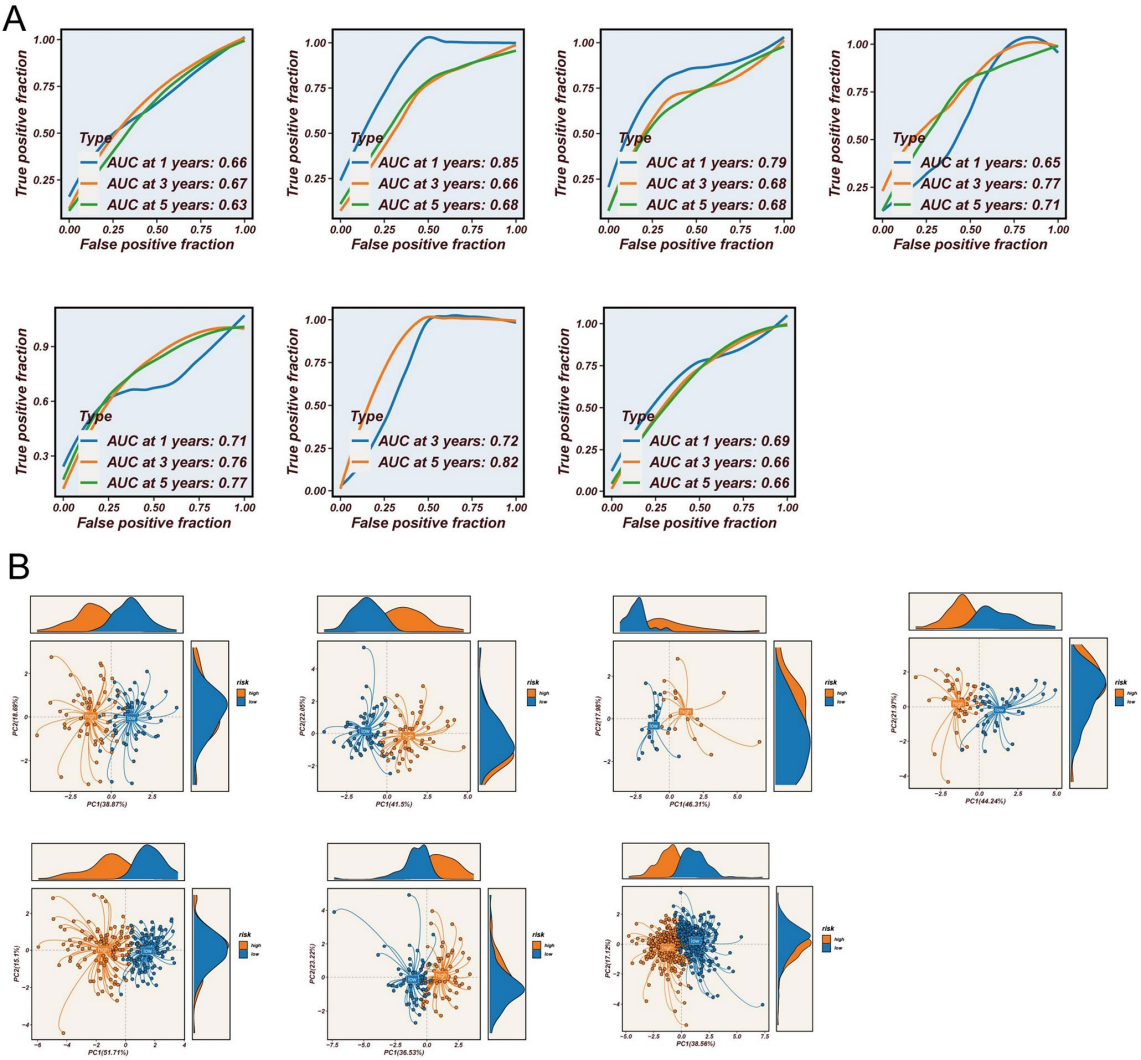
Prediction value. (A) ROC curve for 1‐year, 3‐year, and 5‐year survival predictions. (B) PCA of high‐risk samples and low‐risk samples across seven datasets.

**FIGURE 7:**
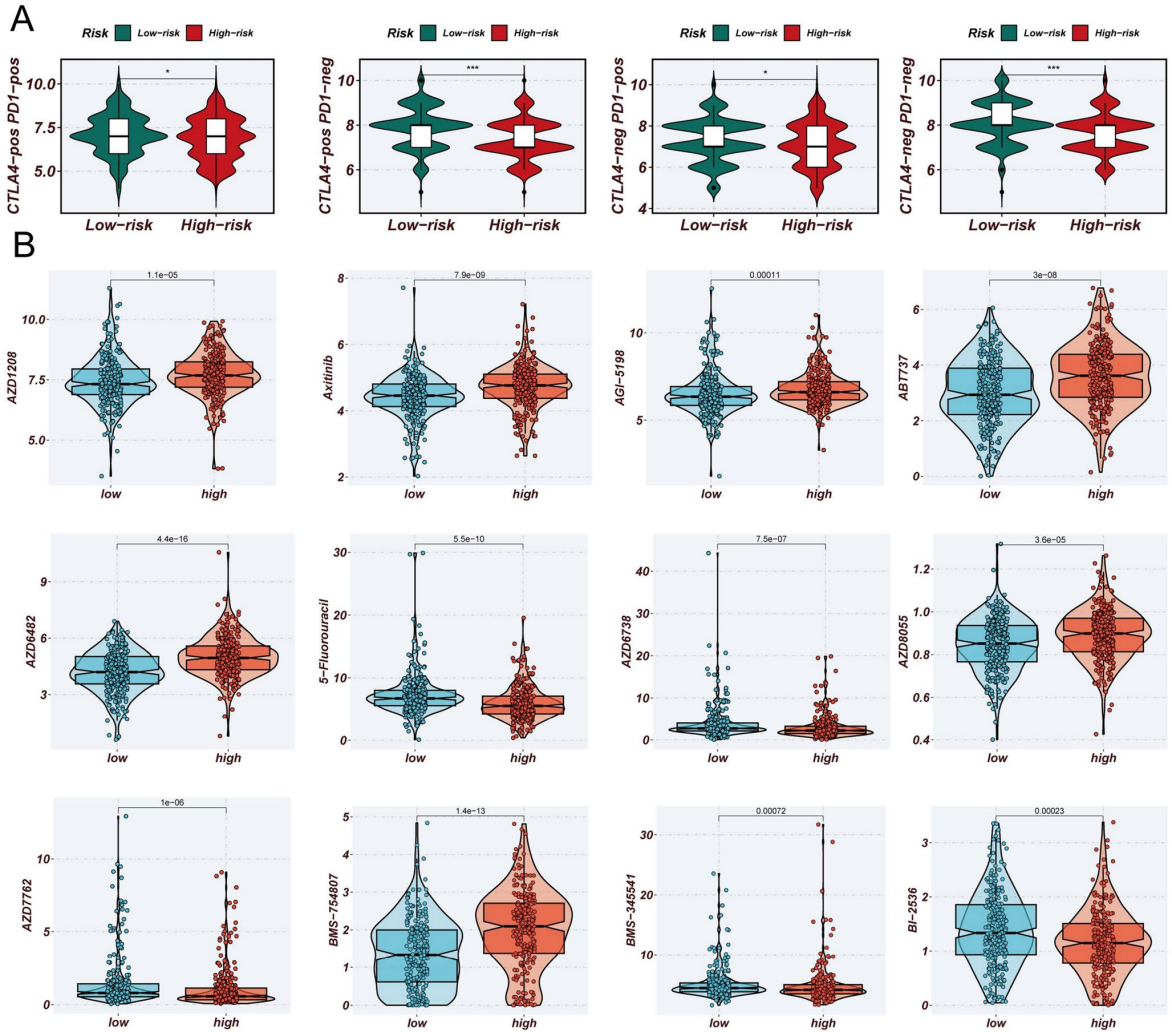
Prediction value of chemotherapy and immune microenvironment. (A) Prediction of IPS for TCGA‐LUAD patients using The Cancer Immunome Atlas, consistently indicating higher IPS and greater sensitivity to immunotherapy in the low‐risk group. (B) Prediction of different chemotherapy for low‐risk and high‐risk group.

Utilizing the expression profiles of prognosis‐related ac4C genes, an ARGSig was developed via an ensemble of machine learning algorithms. The TCGA dataset served as the training cohort, while six GEO datasets were employed for validation. The average C‐index across the six validation cohorts guided model selection, ultimately identifying the combination of RSF and survival support vector machine (survivalSVM) as optimal (Figure 5A). ARGSig scores stratified patient prognosis effectively across all seven cohorts (Figure 5B), with high‐ARGSig patients exhibiting poorer survival outcomes compared to low‐ARGSig counterparts. ROC curves confirmed the robust prognostic predictive capability of ARGSig, consistently validated across datasets with 1‐, 3‐, and 5‐year AUC values typically exceeding 0.65 (Figure 6A). Principal component analysis based on ARGSig gene expression further demonstrated clear separation between high‐ and low‐risk LUAD patients, indicating distinct clustering patterns (Figure 6B). Moreover, immunophenoscores (IPS) derived from the TCIA database suggested that patients in the low‐ARGSig group are more likely to benefit from PD‐1, CTLA‐4, or combination checkpoint inhibitor therapies (Figure 7A). Chemotherapy sensitivity predictions indicated enhanced responsiveness of the low‐ARGSig group to agents such as 5‐Fluorouracil, AZD6738, and AZD7762 (Figure 7B). Collectively, these findings substantiate ARGSig as a reliable prognostic tool for LUAD and highlight its potential utility in guiding personalized immunotherapeutic and chemotherapeutic interventions.

## 4 Discussion

Significant advancements have been made in the field of epigenetics concerning RNA acetylation, particularly NAT10‐mediated ac4C modification [32, 33]. Beyond its involvement in various physiological processes, ac4C contributes to cancer progression by modulating mRNA stability, thereby influencing cellular proliferation, metastasis, lipid metabolism, and drug resistance [15, 34, 35, 36]. In this study, scRNA‐seq revealed heterogeneous cellular clusters within lung adenocarcinoma, including Epithelial, Macro_FABP4, T cells, B/Plasma, and CD8_NK_like populations, underscoring the complexity of the tumor microenvironment. Notably, ac4C modification levels were markedly elevated in Macro_IFI27 cells compared to other cell types. Cell–cell communication analyses demonstrated that cells in the high‐ac4C group exhibited stronger intercellular interactions, suggesting that ac4C‐associated genes may facilitate enhanced cellular communication. Further information flow analysis identified increased expression of PECAM1, EGF, VISFATIN, ANNEXIN, and LAMININ in the high‐ac4C group, molecules known to play critical roles in tumor initiation, progression, and metastasis [37, 38, 39, 40, 41]. These factors predominantly regulate tumor cell proliferation, migration, immune evasion, angiogenesis, and metabolic reprogramming. Machine learning (ML) has emerged as a powerful tool for extracting meaningful insights from large‐scale datasets. Employing an ensemble of 101 ML algorithm combinations, we developed the ARGSig, which demonstrated superior performance in predicting OS compared to previously reported prognostic signatures. Validation across six independent cohorts further confirmed ARGSig's robust predictive capability, highlighting its potential utility in clinical practice.

Immunotherapy has fundamentally transformed the therapeutic landscape of lung adenocarcinoma, particularly through ICIs such as PD‐1 and PD‐L1 blockers that disrupt tumor immune evasion mechanisms [10, 42, 43]. However, not all patients derive benefit, emphasizing the critical need for precise biomarkers to identify likely responders [44, 45, 46]. Our analysis revealed significant differences in immune status between the two ac4C‐based risk groups. The low‐risk group exhibited higher levels of immune cell infiltration, suggesting enhanced responsiveness to immunotherapy. Additionally, predictions of chemotherapeutic response indicated greater sensitivity of the low‐ARGSig group to agents including 5‐Fluorouracil, AZD6738, and AZD7762, underscoring ARGSig's potential as a biomarker for both immunotherapeutic and chemotherapeutic efficacy. These findings support ARGSig as a valuable tool for stratifying lung adenocarcinoma patients most likely to benefit from these treatments.

The ARGSig developed herein exhibits strong prognostic relevance, correlating significantly with patient outcomes and thereby underscoring its promise as a clinical biomarker. Nonetheless, several limitations warrant consideration. First, this study focused exclusively on lung adenocarcinoma to reflect ac4C modification activity at the single‐cell level; other NSCLC subtypes, such as squamous cell carcinoma, were not addressed. Future studies should investigate these subtypes as suitable datasets become available. Second, the ARGSig gene list may be incomplete, which could introduce bias. Lastly, the prognostic value of ARGSig requires validation in larger, multicenter cohorts. To further validate the clinical applicability of ARGSig, prospective studies and wet‐lab experiments are warranted. Prospective cohorts could be designed to prospectively collect samples from lung adenocarcinoma patients and evaluate the predictive accuracy of ARGSig in real‐time clinical settings. Additionally, wet‐lab experiments, such as knockdown or overexpression studies of key genes within the ARGSig, could elucidate the underlying biological mechanisms and confirm the functional relevance of these genes in tumor progression and therapeutic response. Such validation efforts would provide robust evidence for the integration of ARGSig into clinical decision‐making processes. Despite these limitations, this work provides critical insights into the role of ac4C in lung adenocarcinoma and presents a novel prognostic framework. The ARGSig model not only facilitates personalized therapeutic guidance but also identifies new targets and directions for future LUAD treatment strategies.

## 5 Conclusion

This study demonstrates that ac4C modification plays a vital role in lung adenocarcinoma progression. Using transcriptomic and single‐cell data, we identified cellular differences in ac4C activity linked to tumor‐related pathways and cell communication. The ARGSig developed through machine learning showed strong prognostic value and effectively predicted patient responses to immunotherapy and chemotherapy. These results suggest ARGSig as a useful biomarker for personalized treatment decisions. Future studies should validate this model in larger cohorts and explore its applicability to other lung cancer subtypes. Overall, this work provides new understanding of ac4C's role and offers a tool to improve lung adenocarcinoma patient management.

## Author Contributions

Yuan Wang, Wei Su, and Guangyao Zhou contributed equally as co‐first authors, making substantial contributions to data collection, analysis, and manuscript drafting. Yijie Wang assisted in data processing and analysis. Chunnuan Wu supported the research work. Chunnuan Wu, Pengpeng Zhang, and Lianmin Zhang served as co‐corresponding authors, providing overall research design, academic guidance, and critical manuscript revision, offering significant oversight to the research's academic quality and direction.

## Conflicts of Interest

The authors declare no conflicts of interest.

## Data Availability

This study used public data from online database. The data can be obtained freely from TCGA (https://www.cancer.gov/ccg/research/genome‐sequencing/tcga) and GEO (https://www.ncbi.nlm.nih.gov/geo/).
